# Paraneoplastic podocytopathies with nephrotic syndrome in solid cancers with C-Maf inducing protein expression

**DOI:** 10.1093/ckj/sfag370

**Published:** 2026-10-15

**Authors:** Arthur Michon Colin, Shao-Yu Zhang, Vincent Audard, David Buob, Dimitri Titeca-Beauport, Henri Brenier, Evangeline Pillebout, Céline Lebas, Nizar Joher, Damien Sene, Alexandre Karras, Mohamad Zaidan, Sophie De Seigneux, Arwa Jalal Eddine, Dil Sahali, Jean-Jacques Boffa

**Affiliations:** Service de Physiologie—Explorations Fonctionnelles, Hôpital Européen Georges Pompidou, Assistance Publique—Hôpitaux de Paris, Paris, France; Université Paris-Est Créteil, INSERM U955, Institut Mondor de Recherche Biomédicale, Créteil, France; Université Paris-Est Créteil, INSERM U955, Institut Mondor de Recherche Biomédicale, Créteil, France; Service Néphrologie et Transplantation, Hôpitaux Universitaires Henri Mondor, Centre de Référence Maladies rares « Syndrome Néphrotique Idiopathique », Assistance Publique—Hôpitaux de Paris, Créteil, France; Sorbonne Université, INSERM U1155, Hôpital Tenon—Bâtiment Recherche, Paris, France; Service d’Anatomie et cytologie pathologiques, Hôpital Tenon, Assistance Publique - Hôpitaux de Paris, Paris, France; Service de Néphrologie, CHU Amiens Picardie (site Sud), Amiens, France; Service de Néphrologie, CHU Pontchaillou, Université de Rennes, Rennes, France; Service de Néphrologie et Transplantation, Hôpital Saint-Louis, Assistance Publique—Hôpitaux de Paris, Paris, France; Service de Néphrologie et Transplantation rénale, Centre Hospitalier Régional Universitaire de Lille, Lille, France; Service Néphrologie et Transplantation, Hôpitaux Universitaires Henri Mondor, Centre de Référence Maladies rares « Syndrome Néphrotique Idiopathique », Assistance Publique—Hôpitaux de Paris, Créteil, France; Service de Médecine Interne, Hôpital Lariboisière, Assistance Publique—Hôpitaux de Paris, Paris, France; Service de Néphrologie, Hôpital Européen Georges Pompidou, Assistance Publique—Hôpitaux de Paris, Paris, France; Service de Néphrologie, Hôpital Bicêtre, Assistance Publique—Hôpitaux de Paris, Le Kremlin-Bicêtre, France; Université Paris Saclay, INSERM U1184, Le Kremlin-Bicêtre, France; Service de Néphrologie, Hôpitaux universitaires de Genève, Genève, Switzerland; Service de Néphrologie, Hôpital Foch, Suresnes, France; Université Paris-Est Créteil, INSERM U955, Institut Mondor de Recherche Biomédicale, Créteil, France; Service Néphrologie et Transplantation, Hôpitaux Universitaires Henri Mondor, Centre de Référence Maladies rares « Syndrome Néphrotique Idiopathique », Assistance Publique—Hôpitaux de Paris, Créteil, France; Sorbonne Université, INSERM U1155, Hôpital Tenon—Bâtiment Recherche, Paris, France; Service de Néphrologie et Dialyse, Hôpital Tenon, Assistance Publique—Hôpitaux de Paris, Paris, France

**Keywords:** C-Maf inducing protein, focal segmental glomerulosclerosis, minimal change disease, nephrotic syndrome, paraneoplastic glomerulopathy

## Abstract

**Background:**

The main features of minimal change disease (MCD) and focal segmental glomerulosclerosis associated with solid cancer [paraneoplastic podocytopathy with nephrotic syndrome (PPNS)] remain poorly described.

**Methods:**

In this French retrospective study, we followed up patients with PPNS and compared them to a control group with idiopathic nephrotic syndrome (INS). Demographic, clinical, laboratory, and pathological data were collected for both groups. Expression of C-Maf inducing protein (CMIP) was studied on kidney biopsies and tumor samples from PPNS and controls (patients with INS or cancer without nephrotic syndrome).

**Results:**

Twenty-one PPNS patients (mean age 68 years, 76% men, 76% MCD) were identified. Baseline biological characteristics in PPNS (median urinary protein to creatinine ratio 0.87 g/mmol, albuminemia 15 g/l, and glomerular filtration rate 48 ml/min/1.73 m²) and INS (0.90 g/mmol, 15.3 g/l, and 60 ml/min/1.73 m², respectively) patients were similar. Complete remission of nephrotic syndrome occurred less frequently in PPNS than in INS (25% vs 74%, *P* = .008).

CMIP was highly detected on podocytes from PPNS and INS, and in tumors from PPNS (13/13 samples, either in tumor cells and/or in lymphocytic infiltrates), but its expression was scarce in tumors not associated with nephrotic syndrome. Immunofluorescence analysis revealed that CMIP overexpression was confined to a CD4⁺ FOXP3⁻PD1⁻ T-cell subpopulation.

**Conclusions:**

Initial presentation of PPNS is similar to that of INS but complete remission is less frequent. The tight association between nephrotic syndrome and CMIP expression in tumor cells suggests that CMIP might participate in this paraneoplastic syndrome.

## Graphical Abstract

**Graphical Abstract:**
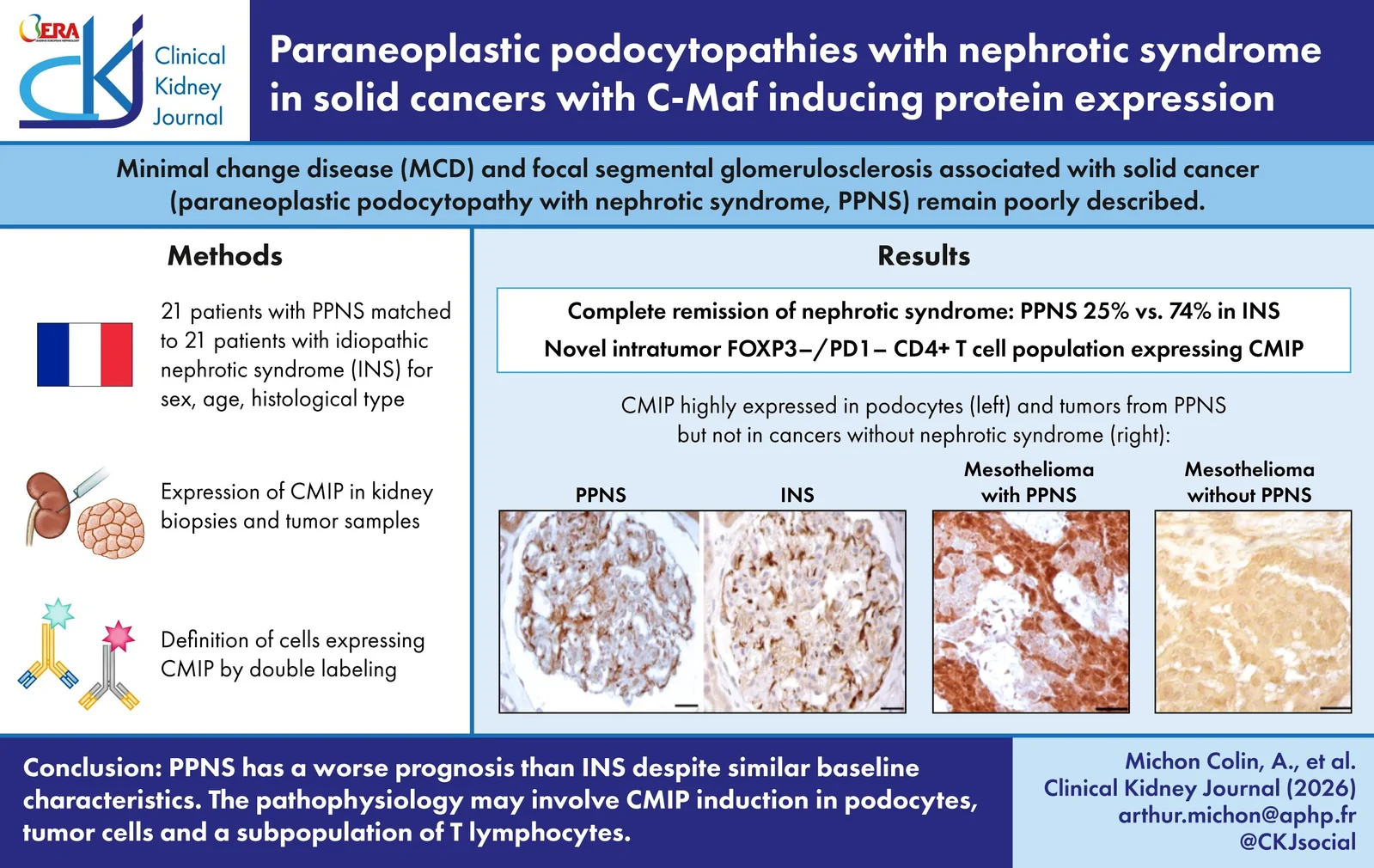

KEY LEARNING POINTS **What was known:**

- Paraneoplastic nephrotic syndrome associated with solid cancers is classically linked to membranous nephropathy, whereas minimal change disease (MCD) and focal segmental glomerulosclerosis (FSGS) have only been reported in isolated case reports and small series.
- The clinical presentation, outcomes, and pathological features of MCD/FSGS occurring in patients with solid cancers remain poorly characterized, making the significance of this association uncertain.
- C-Maf inducing protein (CMIP) has been implicated in idiopathic nephrotic syndrome and Hodgkin lymphoma-associated MCD, but its role in podocytopathies associated with solid tumors was unknown.

**This study adds:**

- This multicenter study provides the largest series to date of paraneoplastic podocytopathy with nephrotic syndrome (PPNS) associated with solid cancers, showing that its initial presentation closely resembles idiopathic nephrotic syndrome.
- Despite similar baseline nephrotic syndrome severity, patients with paraneoplastic podocytopathy achieved complete remission less frequently than matched patients with idiopathic nephrotic syndrome.
- CMIP was consistently expressed in podocytes and in tumors from affected patients, but not in tumors without nephrotic syndrome, and identified a FOXP3^−^/PD1^−^CD4^+^ T-cell subset that may participate in disease pathogenesis.

**Potential impact:**

- Kidney biopsy should be considered in patients with solid cancer and nephrotic syndrome, as paraneoplastic MCD or FSGS may be more common than previously recognized.
- Distinguishing PPNS from idiopathic nephrotic syndrome may improve prognostic assessment and facilitate integrated oncological and nephrological care.
- Identification of CMIP expression in tumors, podocytes and infiltrating T cells provides a rationale for future biomarker-driven and mechanistic studies in cancer-associated nephrotic syndrome.

## INTRODUCTION

Idiopathic nephrotic syndrome (INS) is a primary glomerular disease, which mainly includes two histological variants, minimal change disease (MCD) and focal segmental glomerulosclerosis (FSGS). Both entities result from damage to the epithelial cells lining the glomerular basement membrane called podocytes, leading to the designation podocytopathy [1]. Kidney biopsies hardly show inflammation lesions, immune cell infiltrates, immunoglobulin, and complement deposits. Clinical and experimental evidence strongly suggests that both immune system and podocyte dysfunction are two sides of the disease, which is thought to be mediated by one or more putative circulating factors that increase glomerular capillary permeability leading to induced podocyte dysfunction and subsequent proteinuria [1−3]. The association between MCD and lymphoid disorder such as Hodgkin and non-Hodgkin lymphoma or thymoma supports the hypothesis that this glomerular disease results from an immune system disorder [4−6]. The presence of several autoantibodies, including anti-nephrin antibodies [3], in MCD and FSGS patients support the immune disease. Although the pathophysiological relationship remains poorly understood, it has been suggested that malignant or lymphoid tumor cells could secrete certain mediators (hormones, cytokines, growth factors, etc.), which can impair podocyte function; thus this disease is called paraneoplastic nephrotic syndrome (PNS) [7, 8]. PNS occurring in a context of solid cancers is a well-known cause of secondary membranous nephropathy [9], another glomerular disease characterized by subepithelial immune-complex deposition but remains an exceptional finding in patients with biopsy-proven INS [10−15]. Despite the well-described association of INS with lymphoid neoplasm, the main clinical and pathological characteristics of patients with INS occurring in a context of malignant solid tumor have not been studied. This study aimed to characterize the pathologic and clinical characteristics of INS occurring in patients with solid cancer to reassess the significance of this association, which we call paraneoplastic podocytopathy with nephrotic syndrome (PPNS). Moreover, the demonstration of C-Maf inducing protein (CMIP) specific induction in both podocyte and Reed–Sternberg cells of Hodgkin’s lymphoma in patients with MCD and Hodgkin lymphoma [16] led us to investigate its potential expression on kidney biopsy specimen and tumoral tissue in these patients, which may provide new insights in the pathophysiology of this association.

## MATERIALS AND METHODS

### Study population

We conducted a multicenter retrospective study of patients with PPNS. The inclusion criteria were: age >18 years; presence of a histologically proven solid cancer; occurrence during cancer follow-up or concomitant discovery (±3 months) of a nephrotic syndrome (NS): proteinuria >3 g/24 h or urinary protein to creatinine ratio (UPCR) >0.3 g/mmol predominantly composed of albumin, with serum albumin <30 g/l or proteinemia <60 g/l. INS diagnosis consisted in the pathological features of MCD or FSGS on renal biopsy specimen. Non-inclusion criteria were the presence of another cause of NS and the presence of a hematological disease.

Clinical and biological characteristics, therapeutic management, and outcome of PPNS patients were compared to a control group of INS matched for sex, age (±5 years), and histological type (MCD or FSGS). Of note, control patients over 60 years old were all screened according to the standard of care to exclude a cancer associated with NS.

### Data collection

Baseline demographic, clinical, biological, and histological data were collected for all PPNS patients and patients of the INS control group at the time of renal biopsy; after 3, 6, and 12 months of follow-up; and at the last visit.

Complete remission of NS was defined as urinary protein to creatinine ratio (UPCR) <0.05 g/mmol combined with serum albumin concentrations >30 g/l. Partial remission of NS was defined as persistent proteinuria, UPCR >0.05 g/mmol but with an albumin level >30 g/l up. Complete remission of malignancy was assessed by the referring oncology team.

For each patient, we performed immunohistochemical labeling of CMIP on samples of both kidney biopsy and malignant tissue. In addition, a similar analysis was performed on kidney biopsies from INS control patients and patients with matched type of neoplasia but without diagnosis of NS.

### Immunohistochemistry and confocal microscopy

Paraffin-embedded sections were processed using standard immunohistochemical procedures. Tissue sections were incubated overnight with anti-CMIP antibody (2.5 μg/ml). Detection was performed using an avidin–biotin peroxidase system followed by hematoxylin counterstaining.

For immunofluorescence on paraffin sections, the primary antibodies used in this study include monoclonal rabbit anti-CD3, anti-CD8, anti-FoxP3, anti-CD4, PD-1, and CD20. The mouse monoclonal anti-CMIP (8D5) was developed by our laboratory (antibody specificity data available in Fig. S1). Indicated antibodies were stained on these sections overnight. Then, slides were simultaneously incubated with fluorescent secondary antibodies and mounted with DAPI-containing medium. Negative controls were stained with universal IgG isotype control instead of primary antibodies. Tissue sections were imaged by a confocal laser scanning microscope LSM510-META (Carl Zeiss, Germany). For quantification, all glomeruli from each section, except those with significant sclerosis, were analyzed by computer-assisted image analysis using 400× magnification. The images were blindly analyzed by two independent investigators, as previously described [17]. Positive staining within each glomerulus was expressed as the percentage of the immunostained area over the total glomerular area using the image analysis software (Image J [17, 18]; National Institute of Health, Bethesda, MD). Detailed methods for immunohistochemistry and immunofluorescence analyses are provided in the Supplementary material.

### Patient consent

All patients received oral and written information and agreed to the use of their personal data and residual biopsy according to Helsinki principal and French regulation on retrospective non-interventional medical research [approval of the local institutional review board (IRB 412 Mondor number 00 003 835)] and the Comité de Protection des Personnes d’Ile de France IV (number 2016/25NICB).

### Statistical method

For continuous variables, the median and interquartile ranges at 25% and 75% of values were calculated. Continuous variables of PPNS and controls were compared using a Mann–Whitney test, because of the small sample size, 95% confidence interval (CI) were estimated using a non-parametric bootstrap procedure (2000 resamples with replacement), with CI bounds derived from the 2.5th and 97.5th percentiles of the resampled distribution. The comparison of proportions between PPNS and controls was done by a Fisher’s exact test, 95% CIs were calculated using the Wilson score method. Quantitative analyses of immunohistochemical staining and confocal immunofluorescence images were performed using one-way ANOVA, Fisher’s exact test, or an unpaired *t*-test, as appropriate, as specified in the corresponding figure legends. The *P*-value obtained was considered significant if less than .05.

## RESULTS

### Study population and controls

Twenty-one PPNS patients from 14 French hospitals were identified (between 2014 and 2022 in 19 cases). Characteristics of the study population at the time of renal biopsy are summarized in Table 1. Median age of PPNS patients was 68 years [IQR 25%–75%: 62–74] and 24% of them were women. PPNS was associated with a broad spectrum of malignancies, most commonly lung (*n* = 6), colon (*n* = 3), kidney (*n* = 3), and prostate (*n* = 3) cancers, followed by mesothelioma, pancreatic, endometrial, and ovarian cancers; cholangiocarcinoma; and cancer of unknown primary origin (*n* = 1 each). The two conditions were diagnosed concurrently in 81% of cases (17 out of 21), and only 4 patients (19%) were being diagnosed with cancer for more than 3 months. All patients displayed NS, with a median UPCR at 0.87 g/mmol [IQR 25%–75%: 0.58–1.40] and a median albumin level at 15 g/l [IQR 25%–75%: 11.7–22]. Median glomerular filtration was 48 ml/min/1.73 m² [IQR 25%–75%: 33.3–69]. Renal pathological diagnoses consisted in MCD in 76% and FSGS in 24% of cases. Only four patients underwent ultrastructural analysis (which is not routinely performed in France), which confirmed the MCD diagnosis by showing a diffuse podocytes foot process effacement.

**Table 1:** Patients characteristics at baseline.

	PPNS	*n*	INS (controls)	*n*	*P*
Demography					
Age (years)	68 (62–74)	21	63 (59–73)	21	.30
Women	5 (24 %)	21	5 (24 %)	21	1.00
BMI (kg/m²)	26.9 (24.3–30.4)	20	26.2 (25–29.1)	20	.95
Comorbidity					
Smoking >10 pack-years	14 (70 %)	20	10 (50 %)	20	.33
Pre-existing hypertension	9 (43 %)	21	10 (48 %)	21	1.00
Diabetes	3 (14 %)	21	6 (29 %)	21	.45
HIV	0 (0 %)	21	0 (0 %)	21	1.00
Autoimmune disease	3 (14 %)	21	0 (0 %)	21	.23
Monoclonal gammopathy	2 (10 %)	21	4 (19 %)	21	.66
Clinical and histological characteristics					
Edema	18 (86 %)	21	20 (95 %)	21	.61
Renal pathological findings		21		21	.73
MCD	16 (76 %)		14 (67 %)		
FSGS	5 (24 %)		7 (33 %)		
Renal histological lesions		20		21	
% sclerotic glomeruli	11 (0–16)		0 (0–16)		.41
% IFTA^a^	10 (0–11)		5 (0–10)		.32
Neoplasia type		21			
Bronchopulmonary cancer	6 (29 %)				
Prostatic adenocarcinoma	3 (14 %)				
Clear cell renal carcinoma	3 (14 %)				
Colorectal cancer	3 (14 %)				
Other: mesothelioma, pancreas, endometrium, ovary, cholangiocarcinoma, unknown	6 (29 %)				
Metastatic stage	12 (57 %)	21			
Anti-tumor treatment		21			
Surgery alone	6 (29 %)				
Radiotherapy	5 (24 %)				
Chemotherapy	11 (52 %)				
Targeted treatment/hormone therapy	6 (29 %)				
Specific treatment of nephrotic syndrome	14 (67 %)	21	21 (100 %)	21	.009
Biological analysis					
GFR according to CKD-EPI (ml/min/1.73 m²)^b^	48 (33–69)	20	60 (47–87)	21	.35
Albumine level (g/l)	15 (11.7–22)	21	15.3 (12–21.6)	21	.97
Hemoglobin level (g/dl)	12 (10.2–13.1)	21	13.4 (12.1–14.2)	20	.039
Leukocytes count (G/l)	7.7 (5.9–10.1)	20	6.4 (5.5–7.5)	20	.091
Platelets count (G/l)	290 (234–406)	21	261 (217–305)	19	.30
UPCR (g/mmol creatinine)	0.87 (0.58–1.40)	21	0.9 (0.67–1.12)	21	.70
Hematuria^c^	9 (45 %)	20	3 (14 %)	21	.043

Note: All continuous variables are presented as median + interquartile range (IQR25%–IQR75%). All proportions are presented as number of patients + %.

Abbreviations: BMI, body mass index; FSGS, focal segmental glomerulosclerosis; HIV, human immunodeficiency virus; IFTA, interstitial fibrosis/tubular atrophy; INS, idiopathic nephrotic syndrome; MCD, minimal change disease; PPNS, paraneoplastic podocytopathy with nephrotic syndrome; UPCR, urinary protein to creatinine ratio.

^a^IF/TA, counted at 10% if “discrete/moderate”, at 5% if “<10%” or “minimal”, side 0 if “no fibrosis”.

^b^Estimate glomerular filtration rate (eGFR) according to the CKD-EPI formula (in ml/min/1.73 m²), set at 0 ml/min/1.73 m² in case of hemodialysis.

^c^Hematuria >20.000/ml, leukocyturia >10.000/ml, or positivity on urine strip test if data not available.

At presentation, PPNS patients more frequently exhibited microscopic hematuria (*P* = .04) and a lower median hemoglobin level (*P* = .04) than INS controls. Moreover, PPNS was less often treated with immunosuppressive drugs. All PPNS patients received cancer-directed therapy, but only 14 out of 21 (67%) also received specific immunosuppressive therapy (compared with 100% of INS controls treated by first line steroid therapy, *P* = .004), which consisted of steroids alone for all patients except one who received an anti-CD20 monoclonal antibody in association with steroids therapy.

### Follow-up

PPNS patients had a less favorable significant outcome than controls in terms of NS remission after 3, 6, and 12 months of follow-up (Table 2). After 12 months, only 3 PPNS patients out of 12 (25%) achieved complete remission, compared to 14 out of 19 INS controls (74%) (*P* = .012). Considering global remission (complete and partial), the difference persisted but did not reach significance with 9 PPNS patients out of 12 (75%) versus 18 out of 19 controls (95%, *P* = .27). We did not find any statistical difference regarding renal function outcome between PPNS and the control group at any time of the follow-up (Table 2). At 12 months, the mortality rate was three-fold higher among PPNS patients than in the control group (30% vs. 10%, *P* = .13). Given the risk of informative censoring related to this excess mortality, a sensitivity analysis was performed. The PPNS patients who died before M12 (or whose vital status at M12 was unknown) were conservatively reclassified as treatment failures. This sensitivity analysis confirmed the difference in complete remission rates between PPNS and INS (PPNS 3/21 [14.3%] vs. INS 14/21 [66.7%], *P* = .0013). The outcome of PPNS was analyzed according to oncological response. Among four patients in complete oncologic response, two were in complete remission and one in partial remission of NS. The three patients with partial oncologic response were also in partial NS remission. Of the 14 patients with unfavorable oncologic evolution, 7 (50%) showed no improvement in their NS. However, four (29%) had a complete remission of NS despite cancer progression. Thus, PPNS renal outcome was mostly correlated with oncologic outcome.

**Table 2:** Follow-up data for PPNS and INS.

	PPNS	*n*	INS (control group)	*n*	*P*
Clinical evolution					
Complete remission of NS					
M3	3 (14 %) [95% CI 5.0%–34.6%]	21	10 (48 %) [95% CI 28.%3–67.6%]	21	.043
M6	3 (16 %) [95% CI 5.5%–37.6%]	19	10 (53 %) [95% CI 31.7%–72.7%]	19	.038
M12	3 (25 %) [95% CI 8.9%–53.2%]	12	14 (74 %) [95% CI 51.2%–88.2%]	19	.012
Last visit	6 (29 %) [95% CI 13.8%–50.0%]	21	16 (76 %) [95% CI 54.9%–89.4%]	21	.0048
Global (partial + complete) remission of NS					
M3	8 (38%) [95% CI 20.8%–59.1%]	21	13 (62%) [95% CI 40.9%–79.2%]	21	.22
M6	12 (63%) [95% CI 41.0%–80.9%]	19	14 (74%) [95% CI 51.2%–88.2%]	19	.73
M12	9 (75%) [95% CI 46.8%–91.1%]	12	18 (95%) [95% CI 75.4%–99.1%]	19	.27
Last visit	13 (62%) [95% CI 40.9%–79.2%]	21	20 (95%) [95% CI 77.3%–99.2%]	21	.020
Relapse of NS at 12 months^a^	3 (30 %) [95% CI 10.8%–60.3%]	10	5 (29 %) [95% CI 13.3%–53.1%]	17	1.00
Complete remission of cancer					
M3	2 (10 %)	21			
M6	3 (19 %)	16			
M12	3 (27 %)	11			
Last visit	4 (19 %)	21			
Mortality at 12 months	6 (30 %) [95% CI 14.5%–51.9%]	20	2 (10 %) [95% CI 2.7%–28.9%]	21	.13
Biological analysis					
eGFR according to CKD-EPI (ml/min/1.73 m²)					
M0	48 (IQR 33–69) [95% CI 34.0–66.0]	20	60 (IQR 47–87) [95% CI 48.0–83.0]	21	.35
M3	58 (IQR 32–80) [95% CI 32.0–75.0]	19	75 (IQR 61–90) [95% CI 64.0–89.0]	21	.18
M6	60 (IQR 31–77) [95% CI 31.0–77.0]	17	70 (IQR 37–90) [95% CI 40.0–89.5]	18	.45
M12	77 (IQR 29–92) [95% CI 29.0–94.5]	12	72 (IQR 61–89) [95% CI 63.0–87.0]	19	.82
Last visit	71 (IQR 23–84) [95% CI 29.0–83.0]	21	81 (IQR 59–92) [95% CI 64.0–90.0]	21	.17

Note: All continuous variables are presented as median + interquartile range (IQR25%–IQR75%) + 95% confidence intervals (95% CI). All proportions are presented as number of patients + % + 95% confidence intervals (95% CI).

Abbreviations: eGFR, estimated glomerular filtration rate; (I)NS, (idiopathic) nephrotic syndrome; PPNS, paraneoplastic podocytopathy with nephrotic syndrome.

^a^Only patients with at least partial remission were considered.

### Histological findings

We investigated CMIP expression in 13 tumor samples and 16 kidney biopsy specimens from 17 PPNS patients, including 12 tumor/kidney pairs. CMIP expression was also studied in controls, including 5 kidney specimens from patients with INS and 15 tumor samples from patients with cancer but without NS. CMIP was detectable and diffusely distributed along the external side of capillary loops, highly suggestive of podocyte labeling as previously described [17]. Moderate to high intensity podocyte labeling of CMIP was found on all renal biopsies of PPNS except one. Control biopsies of INS also showed clear podocyte CMIP induction (Fig. 1a). The relative abundance of CMIP was quantified in all glomeruli from PPNS and compared with that observed in idiopathic forms (Fig. 1b). CMIP abundance was significantly higher in PPNS and INS relapse than in controls and INS remission. Notably, glomeruli from patients with mesothelioma-associated PPNS exhibited higher CMIP expression than those from patients with INS relapse **(** Fig. 1a).

**Figure 1:**
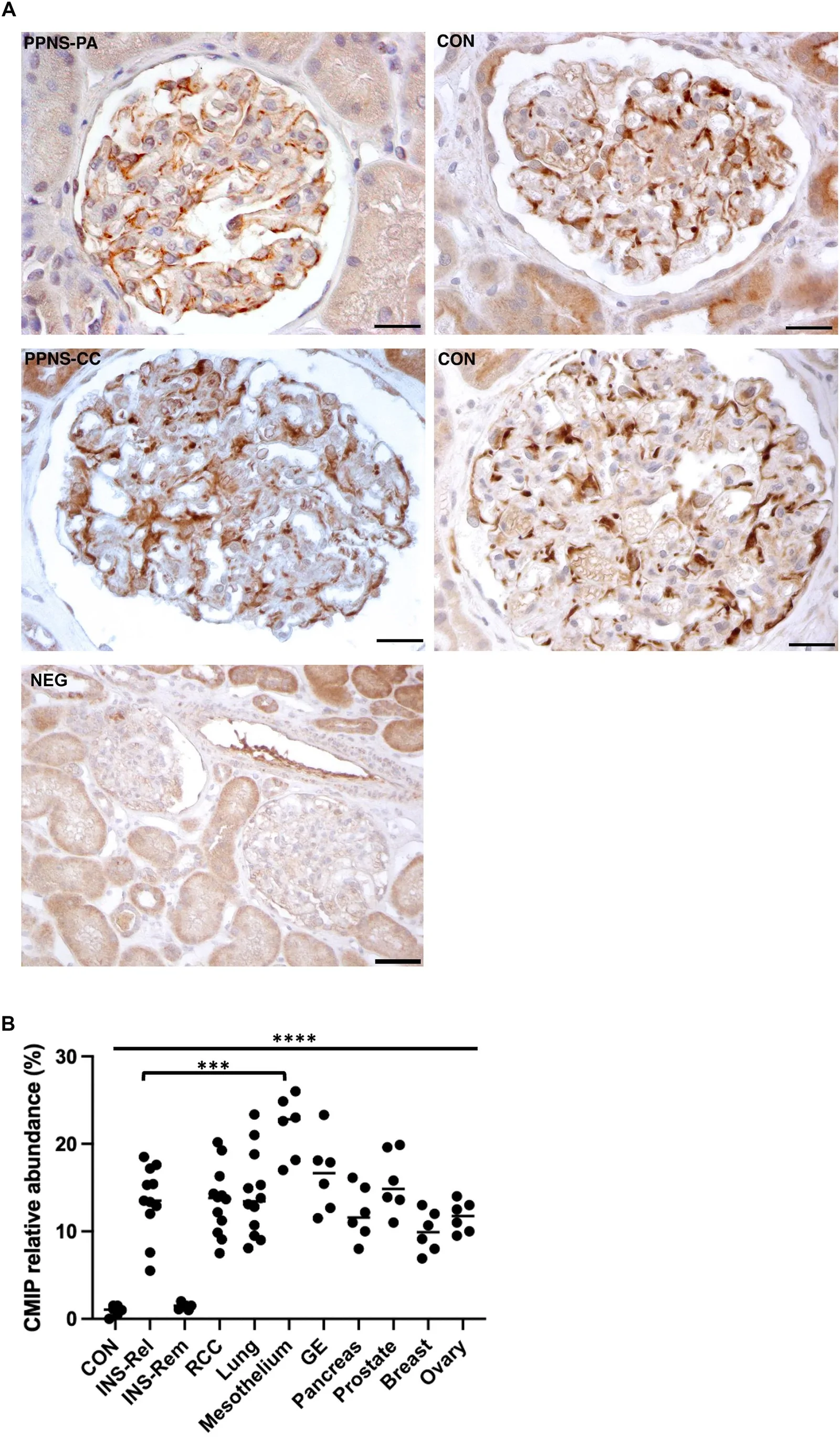
a. Immunohistochemistry of CMIP on histological sections of kidneys from patients with PPNS associated with prostate adenocarcinoma (PPNS-PA) or colon carcinoma (PPNS-CC), from patients with INS (CON), and from a control patient without NS (NEG). Scale bar = 50 µm. b. Intensity of CMIP labeling in immunohistochemistry in patients with PNNS. The relative abundance of CMIP was quantified by computer-assisted image analysis at 400× magnification and expressed as the percentage of CMIP-positive area relative to the total glomerular area. CON, control samples (*n* = 5); INS-Rel, idiopathic nephrotic syndrome during relapse (*n* = 5); INS-Rem, idiopathic nephrotic syndrome during remission (*n* = 3). PPNS kidney samples were obtained from patients with PNS associated with the following cancers: renal cell carcinoma (RCC, *n* = 4), lung cancer (*n* = 4), mesothelioma (*n* = 1), gastrointestinal carcinoma (GE, *n* = 2), pancreatic cancer (*n* = 1), prostate cancer (*n* = 2), ovarian cancer (*n* = 1), and breast cancer (*n* = 1). CMIP expression was significantly higher in PPNS samples than in control renal tumors (one-way ANOVA, *P* < .0001). No significant difference was observed between INS relapse and PPNS samples overall, except in mesothelioma-associated PPNS, in which CMIP expression was significantly higher than in INS relapse (unpaired *t*-test, *P* = .0007).

**Figure 2:**
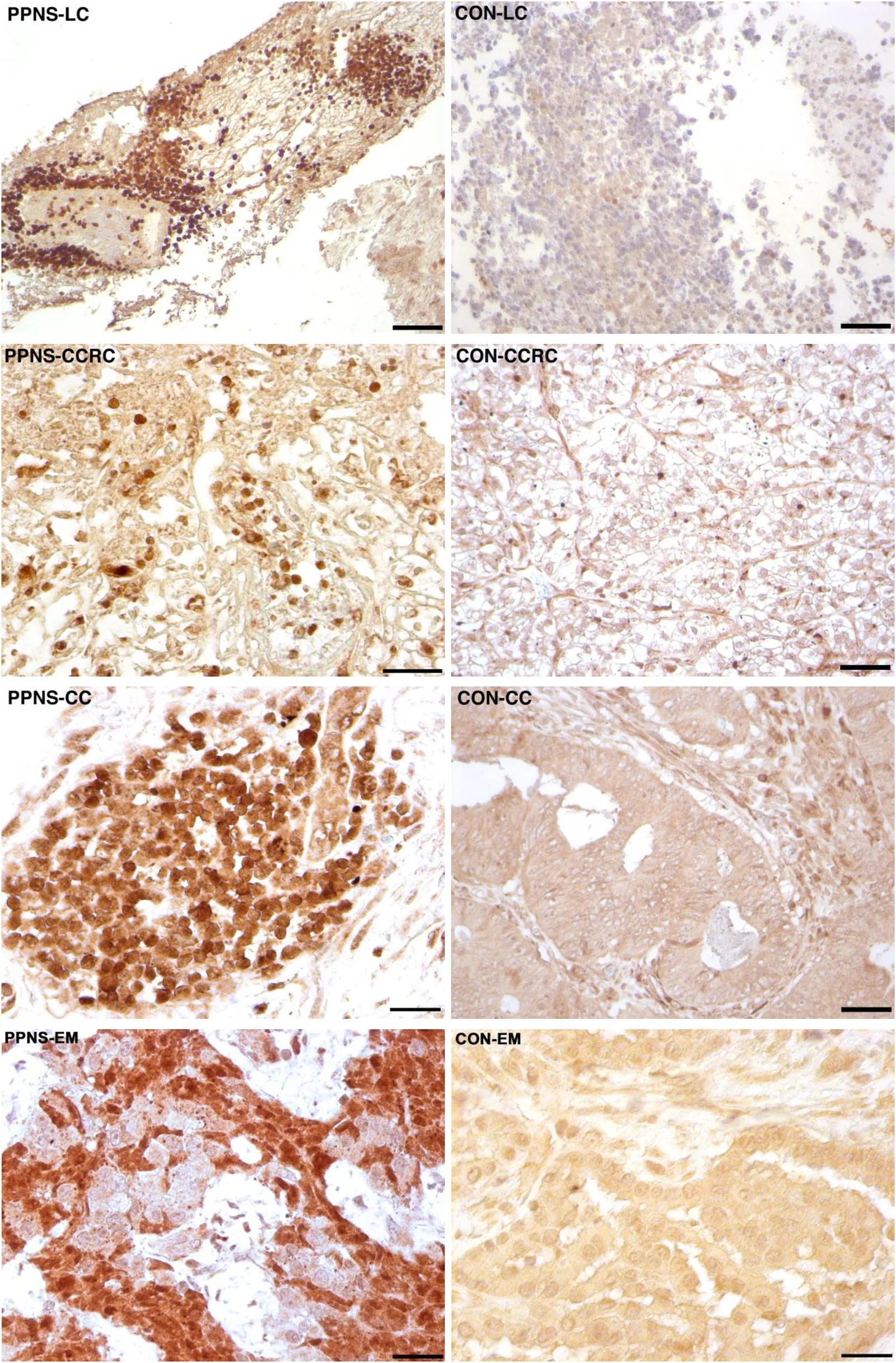
Immunohistochemistry of CMIP on histological sections of cancers from patients with PPNS associated with lung cancer (PPNS-LC), clear cell renal carcinoma (PPNS-CCRC), colon carcinoma (PPNS-CC), or epithelioid mesothelioma (PPNS-EM), and control lung cancer (CON-LC), control clear cell renal carcinoma (CON-CCRC), control colon carcinoma (CON-CC), or epithelioid mesothelioma (CON-EM). Scale bar = 50 µm.).

All available tumor samples from PPNS patients showed moderate to strong CMIP expression in both the cytoplasmic and nuclear compartments, in tumor cells as well as in infiltrating cells (Fig. 2). CMIP staining in tumor and infiltrating cells was heterogeneous. In patients with lung cancer-associated PPNS, the degree of cellular infiltration and number of CMIP-positive cells were higher than in small cell neuroendocrine lung carcinoma. Epithelioid mesothelioma, commonly resulting from asbestos exposure, displayed a particular pattern characterized by massive cytoplasmic expression of CMIP throughout the tumor parenchyma, while CMIP seemed mainly localized in the cytoplasmic compartment of infiltrating cells, to a lesser extent in nuclei. In most other solid tumor-associated PPNS, CMIP was mostly expressed in infiltrating cells. In contrast, CMIP labeling was scarce or undetectable in control tumoral samples (without NS), in favor of PPNS-specific tumor expression of CMIP (Fig. 3).

**Figure 3:**
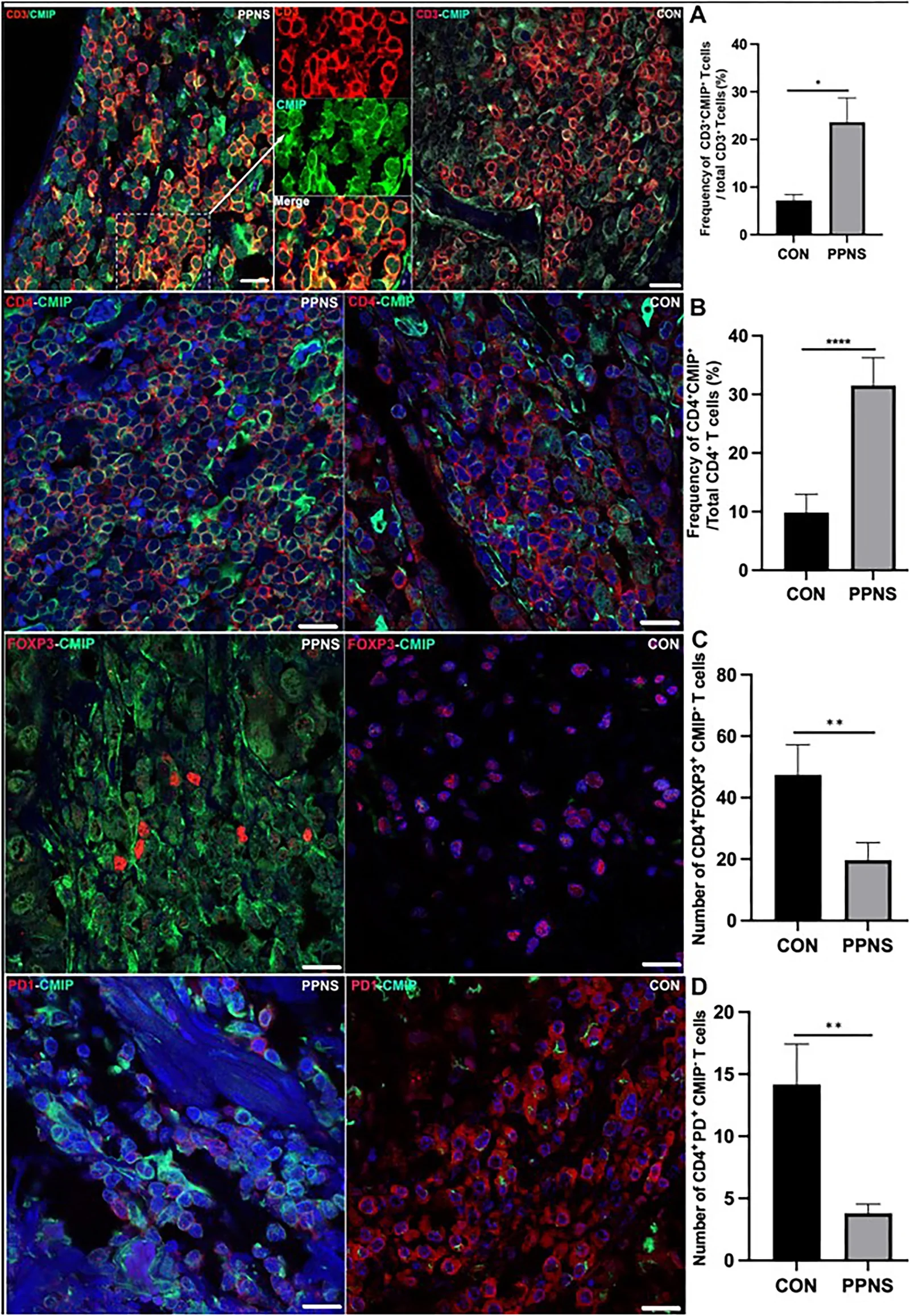
Representative double immunofluorescence staining for CD3/CMIP, CD4/CMIP, FOXP3/CMIP, and PD-1/CMIP, respectively, analyzed by confocal microscopy in samples from patients with PNS associated with cancer (PPNS) and control (CON) samples. Scale bar = 20 µm. (A, B) The proportions of CD3⁺CMIP⁺ T cells among total CD3⁺ T cells and CD4⁺CMIP⁺ T cells among total CD4⁺ T cells were quantified by confocal microscopy in CON and PPNS tumor samples. The frequencies of CD3⁺CMIP⁺ T cells and CD4⁺CMIP⁺ T cells were significantly higher in PPNS tumor samples than in control samples (CD3⁺CMIP⁺ T cells, Fisher’s exact test, *P* = .0113; CD4⁺CMIP⁺ T cells, Fisher’s exact test, *P* < .0001). (C, D) The numbers of CD4⁺FOXP3⁺ T cells and CD4⁺PD-1⁺ T cells were quantified by confocal microscopy in CON and PPNS tumor samples. Notably, CD4⁺FOXP3⁺ T cells and CD4⁺PD-1⁺ T cells were negative for CMIP expression. The numbers of CD4⁺FOXP3⁺ T cells and CD4⁺PD-1⁺ T cells were significantly lower in PPNS tumor samples than in control samples (CD4⁺FOXP3⁺ T cells, Fisher’s exact test, *P* = .0011; CD4⁺PD-1⁺ T cells, Fisher’s exact test, *P* = .0043).

To clarify cellular expression and identity of CMIP positive cells, we carried out double labeling confocal microscopy with an anti-CMIP monoclonal antibody and either an anti-CD3, anti-CD4, anti-CD8, anti-CD20, anti-FoxP3, or anti-PD-1, on four tumor-associated PPNS (lung and colonic adenocarcinoma, clear cell renal carcinoma, and cholangiocarcinoma) for which enough tissue samples were available and on four tumor controls. Initial screening by CMIP/CD3 double labeling showed that only a fraction of T cells was CMIP positive, primarily in the cytoplasmic compartment, while nuclear staining was not clearly evident in all tumor-associated PPNS (Fig. 3). On the other hand, no CMIP expression was detected in the B cell population identified by anti-CD20 antibody, regardless of the degree of B cell infiltrates (Fig. S2). Within the CD3 T cells population, CMIP was mostly expressed in a subset of CD4^+^ T cells, and to a lesser extent in the CD8^+^ T cell population (Fig. 3 and Fig. S2). We next investigated whether FOXP3, a canonical marker of regulatory T cells (Tregs), was differentially expressed in PPNS and control tissues and whether its expression overlapped with CMIP in CD4^+^ T cells. The frequency of CD4^+^FOXP3^+^ T cells was significantly lower in PPNS infiltrates than in control tissues (Fig. 3). Notably, CMIP and FOXP3 expression were mutually exclusive, with CMIP and FOXP3 confined to separate CD4^+^ T-cell subsets. CMIP expression by the CD4^+^PD1^+^T cell subset was investigated, but no CMIP/PD1 co-expression in infiltrating CD4^+^T cells was found. Despite the limited tissue sample size, these findings suggest that CMIP expression is restricted to a subset of infiltrating CD4^+^ T cells with a distinct phenotype that remains to be fully characterized.

## DISCUSSION

This is the first-time report of a patient cohort with paraneoplastic podocytopathies related to solid cancer. This is a rare occurrence, as is the previously described association of MCD with Hodgkin’s lymphoma [3]. Overall and renal prognosis was poorer in PPNS than INS patients. We found specific expression of CMIP in tumor and infiltrating cells in patients with PPNS. This expression was scarce or undetectable in control tumors. CMIP was mostly expressed in CD4^+^ lymphocytes.

Our findings support a strong clinical and biological association between solid cancers and MCD/FSGS in PPNS, but do not establish a causal relationship. Several alternative, non-mutually exclusive explanations should therefore be considered. First, a coincidental occurrence of MCD/FSGS cannot be entirely excluded, although this appears less likely given that the incidence of INS does not increase with age [19], in contrast to the age distribution observed in our PPNS cohort. Second, cancer-associated immune dysregulation may contribute to glomerular injury through multiple mechanisms. However, the higher CMIP expression observed in some immune-cell subpopulations infiltrating PPNS tumors, compared with tumor controls, raises the possibility of an association between CMIP expression in immune cells and podocyte injury. The mechanisms underlying this potential relationship remain unknown and warrant further investigation. Third, most patients received both cancer-directed therapies and immunosuppressive treatment, making it difficult to disentangle the respective contributions of these interventions from the natural course of the renal disease.

Our data show that PPNS mostly affects male elderly patients, which differs from the natural incidence of INS [20]. Although initial biological features of NS were comparable between groups, hemoglobin level and rate of hematuria were, respectively, lower and higher in PPNS than in controls. It is well-known that cancer patients have an increased risk of developing anemia [21]. NS with impaired renal function and hematuria had also been described in a cohort of elderly MCD [22]. More importantly, PPNS patients had a more severe renal prognosis with a lower rate of complete remission than old patients with INS.

In contrast, eGFR trajectories did not differ significantly between PPNS and INS at any time point, despite this markedly lower rate of complete NS remission in PPNS. This dissociation between proteinuria remission and glomerular filtration may reflect close combined nephrological and oncological monitoring, a shorter effective follow-up in PPNS related to competing mortality, or a genuine difference in the trajectory of renal function relative to proteinuria in this population; longer follow-up will be needed to determine whether this dissociation persists.

PPNS was associated with diverse solid tumors, most frequently bronchopulmonary cancers (commonly linked to paraneoplastic syndromes in the general population [23]), mostly adenocarcinoma (leading histological type in men over 60 years old [24]). This heterogeneity is consistent with previous case reports [8−13].

PPNS is likely under- and/or misdiagnosed because kidney biopsy is not frequently performed in patients with advanced cancers and poor medical status. Indeed, while the MCD/FSGS association with solid cancers is not classically described, our series is comparable in size to series of MCD associated with Hodgkin’s lymphoma [4], a more widely recognized entity. Considering that the most common cause of cancer-associated NS is membranous nephropathy, physicians are often reluctant to perform kidney biopsy, while a putative diagnosis of membranous nephropathy was anticipated [10].

The diagnosis of paraneoplastic syndrome is generally based on three criteria [7, 25]. First is the temporal correlation between the two diseases. All patients’ NS developed at the onset or during the course of cancer. Second, the course of both pathologies is thought to be correlated under treatment. This criterion was less systematically met in our series, as a probable result of the very low number of oncologic remissions in these patients. Two complete remissions under corticosteroid therapy despite unfavorable oncological outcomes suggest a potential specific effect of corticosteroid likely acting on one of several immunological mechanisms during cancer evolution. However, most PPNS patients not treated with corticosteroids improved their NS, including two complete remissions with oncological treatment alone, supporting a close link between cancer and PPNS. Setting apart the effect of anti-tumoral treatment from that of corticosteroids for patients receiving both treatments remains difficult, as in most reports in the literature [13]. Given the small number of patients, disease severity, and treatment heterogeneity, this study does not provide any evidence regarding the optimal therapeutic management of these patients nor a definitive proof whether renal remission was attributable to cancer-directed therapy. The third criterion is the pathophysiology link between overexpression of CMIP and PPNS. Similar to what has been proposed in Hodgkin’s lymphoma-associated MCD [16], we explore here the hypothesis that CMIP induction plays a role in the pathogenesis of PPNS.

CMIP is an 86 kDa protein whose exact function is unknown. The CMIP gene was initially discovered in circulating lymphocytes of patients with MCD, by subtractive cloning and differential between the disease flare and remission [26]. CMIP overexpression has since been found in INS with MCD or FSGS, in primary and lupus-associated membranous nephropathy [27, 28] and in certain drug-induced NS [17, 29, 30]. In contrast, CMIP is not overexpressed if the NS is related to diabetic nephropathy, human immunodeficiency virus -associated nephropathy or immunoglobulin A nephropathy [31]. Selective expression of CMIP in peripheral T cells by targeted transgenesis in mice inhibits T cell activation. CMIP interferes with the early events of T-cell signaling by inhibiting lipid raft clustering, the activation of Src kinases (Fyn and Lck) and ZAP-70 [32]. Transgenic T-cells exhibit a lower proliferative capacity and are less prone to producing cytokines, notably interleukin-2, after stimulation. Moreover, CMIP binds to the P85 subunit of phosphatidylinositol 3(PI3)-kinase and prevents its dissociation from the p110 catalytic subunit, which results in inhibition of PI3-kinase activation and downstream signaling molecules, notably protein kinase B (Akt) [33]. It is interesting to note that Akt activation is inhibited *in vitro* and *in vivo* in T cells overexpressing CMIP [31].

Glomerular expression of CMIP was previously demonstrated in mice and human renal tissue [31]. Immunofluorescence findings also give new insights regarding the nature of CMIP positive infiltrating T cells, even if these analyses were conducted on a subset for exploratory purposes, which was not representative of the entire cohort. First, CMIP is undetectable in Tregs identified by strong nuclear expression of FOXP3 transcription factor, suggesting that CMIP and FOXP3 were confined to two distinct T cell subsets. The reduced frequency of FOXP3⁺ T cells in PPNS tumor infiltrates is consistent with previous reports indicating that INS flares are associated with a decrease in Treg populations and increased CMIP expression [32]. Second, CMIP like PD-1 are recruited to inhibit or prevent T cell activity [32]. Our findings suggest that their expression is mutually exclusive and CMIP and PD-1 belong to two distinct T cell subsets. Moreover, the frequency of PD-1 positive T cells, as evaluated by pathologists is lower (<5%) in examined tissues such as cancerous lung, while that of CMIP positive infiltrating T cells is higher. These observations support the hypothesis that different negative regulatory pathways operate in cancer and tumor microenvironments to adapt its immune strategy and enhance its expansion. The phenotype of CMIP positive T cells is not currently clearly established. Another important aspect is the absence of CMIP expression in B cells, which suggests that cancer-associated PPNS likely involves a disorder of T cell compartment. On the other hand, tumor cell labeling was not found in cancers of control patients. This different tumor expression of CMIP argues in favor of its potential direct pathophysiological role in the development of NS. Furthermore, strong CMIP induction had been found on renal and tumor parenchyma in a recent clinical case of paraneoplastic MCD [11]. Strikingly, as previously demonstrated in Reed–Sternberg cells [16], we observed an induction of CMIP in tumoral cells. The significance of this expression and the role of CMIP in the biology of tumoral cells remain to be determined.

This study is the largest currently published series of MCD/FSGS associated with solid tumors. Given the rarity of this disease, it is not possible to determine the best therapeutic approach or to assess the prognostic impact of CMIP on the progression of cancer and NS [34].

This study has several limitations. First, its retrospective design and relatively small sample size, despite the multicenter recruitment across 14 centers over an extended inclusion period (2014–22), introduce potential heterogeneity in diagnostic workup, treatment strategies, and follow-up. Also, detailed chronology of oncological management could not be systematically retrieved for all patients, as data were collected from nephrology records across multiple centers with heterogeneous documentation of treatment timing. These factors, together with the diversity of tumor types represented, limit our ability to draw conclusions regarding a single, common pathogenic mechanism underlying PPNS. Second, kidney biopsy is not routinely performed in patients with advanced cancer and poor performance status. Consequently, selection bias related to biopsy practices cannot be excluded and may limit the broader applicability of our findings. Third, 12-month follow-up data were unavailable for a substantial proportion of PPNS patients, primarily because of death or loss to follow-up. Given the markedly higher mortality observed in PPNS compared with controls, this raises the possibility of informative censoring. We therefore performed a sensitivity analysis in which missing outcomes were conservatively classified as treatment failures. Fourth, because most patients received cancer-directed therapy and immunosuppression concurrently, we could not fully determine the respective contributions of these treatments to renal outcomes. Testing for anti-nephrin antibodies was not available at time of patient care. Their presence or absence may have provided additional diagnostic and pathophysiological insights that could not be addressed in the present study.

In conclusion, we present the first series of cases exhibiting features of paraneoplastic podocytopathies (MCD and FSGS) associated with solid tumors. Specific CMIP expression by tumoral cells and a peritumoral lymphocytic subpopulation was shown. Further studies are needed to confirm these results and the accurate role of CMIP into PPNS pathogenesis.

## Supplementary Material

sfag370_Supplemental_File

## Data Availability

The data that support the findings of this study are available from the corresponding author upon reasonable request.

## References

[1] Saleem MA. Molecular stratification of idiopathic nephrotic syndrome. Nat Rev Nephrol. 2019;15:750–65. 10.1038/s41581-019-0217-5 31654044

[2] Sahali D, Sendeyo K, Mangier M et al. Immunopathogenesis of idiopathic nephrotic syndrome with relapse. Semin Immunopathol. 2014;36:421–9. 10.1007/s00281-013-0415-3 PMC5385209 24402710

[3] Hengel FE, Dehde S, Lassé M et al. Autoantibodies targeting nephrin in podocytopathies. N Engl J Med. 2024;391:422–33. 10.1056/NEJMoa2314471 38804512

[4] Audard V, Larousserie F, Grimbert P et al. Minimal change nephrotic syndrome and classical Hodgkin’s lymphoma: report of 21 cases and review of the literature. Kidney Int. 2006;69:2251–60. 10.1038/sj.ki.5000341 16672913

[5] Kofman T, Zhang S-Yu, Copie-Bergman C et al. Minimal change nephrotic syndrome associated with non-Hodgkin lymphoid disorders: a retrospective study of 18 cases. Medicine (Baltimore). 2014;93:350–8. 10.1097/MD.0000000000000206 PMC4602440 25500704

[6] Karras A, de Montpreville V, Fakhouri F et al. Renal and thymic pathology in thymoma-associated nephropathy: report of 21 cases and review of the literature. Nephrol Dial Transplant. 2005;20:1075–82. 10.1093/ndt/gfh615 15788438

[7] Ronco PM. Paraneoplastic glomerulopathies: new insights into an old entity. Kidney Int. 1999;56:355–77. 10.1046/j.1523-1755.1999.00548.x 10411717

[8] Lien YHH, Lai LW. Pathogenesis, diagnosis and management of paraneoplastic glomerulonephritis. Nat Rev Nephrol. 2011;7:85–95. 10.1038/nrneph.2010.171 PMC3058941 21151207

[9] Lefaucheur C, Stengel B, Nochy D et al. Membranous nephropathy and cancer: epidemiologic evidence and determinants of high-risk cancer association. Kidney Int. 2006;70:1510–7. 10.1038/sj.ki.5001790 16941021

[10] Jhaveri KD, Shah HH, Calderon K et al. Glomerular diseases seen with cancer and chemotherapy: a narrative review. Kidney Int. 2013;84:34–44. 10.1038/ki.2012.484 23364518

[11] Bouatou Y, Koessler T, Oniszczuk J et al. Nephrotic syndrome in small cell lung cancer and induction of C-Mip in podocytes. Am J Kidney Dis. 2017;69:477–80. 10.1053/j.ajkd.2016.09.026 28063735

[12] Vishnu Moorthy A. Minimal change glomerular disease: a paraneoplastic syndrome in two patients with bronchogenic carcinoma. Am J Kidney Dis. 1983;3:58–62. 10.1016/S0272-6386(83)80011-0 6869371

[13] Martinez-Vea A, Panisello J, García C et al. Minimal-change glomerulopathy and carcinoma: report of two cases and review of the literature. Am J Nephrol. 1993;13:69–72. 10.1159/000168592 8322844

[14] Singer CRJ, Boulton-Jones JM. Minimal change nephropathy associated with anaplastic carcinoma of bronchus. Postgrad Med J. 1986;62:213–7. 10.1136/pgmj.62.725.213 PMC2418627 3714609

[15] Nakano Y, Yoshida M, Muraki N et al. Prostate cancer associated with minimal change disease: a case report. Glomerular Dis. 2022;2:145–50. 10.1159/000525040 PMC9710312 36751666

[16] Audard V, Zhang S-Yu, Copie-Bergman C et al. Occurrence of minimal change nephrotic syndrome in classical Hodgkin lymphoma is closely related to the induction of c-mip in Hodgkin-Reed Sternberg cells and podocytes. Blood. 2010;115:3756–62. 10.1182/blood-2009-11-251132 PMC2890576 20200355

[17] Izzedine H, Mangier M, Ory V et al. Expression patterns of RelA and c-mip are associated with different glomerular diseases following anti-VEGF therapy. Kidney Int. 2014;85:457–70. 10.1038/ki.2013.344 24067439

[18] McAdoo SP, Bhangal G, Page T et al. Correlation of disease activity in proliferative glomerulonephritis with glomerular spleen tyrosine kinase expression. Kidney Int. 2015;88:52–60. 10.1038/ki.2015.29 PMC4488852 25715120

[19] Vivarelli M, Massella L, Ruggiero B et al. Minimal change disease. Clin J Am Soc Nephrol. 2017;12:332–45. 10.2215/CJN.05000516 PMC5293332 27940460

[20] Fogo AB. Minimal change disease and focal segmental glomerulosclerosis. Nephrol Dial Transplant. 2001;16:74–76. 10.1093/ndt/16.suppl_6.74 11568250

[21] Ludwig H, Van Belle S, Barrett-Lee P et al. The European Cancer Anaemia Survey (ECAS): a large, multinational, prospective survey defining the prevalence, incidence, and treatment of anaemia in cancer patients. Eur J Cancer. 2004;40:2293–306. 10.1016/j.ejca.2004.06.019 15454256

[22] Colliou E, Karras A, Boffa J-J et al. Outcomes of older patients (≥60 years) with new-onset idiopathic nephrotic syndrome receiving immunosuppressive regimen: a multicentre study of 116 patients. J Clin Med. 2019;8:298. 10.3390/jcm8030298 PMC6463053 30832362

[23] Paraschiv B, Diaconu CC, Toma CL et al. Paraneoplastic syndromes: the way to an early diagnosis of lung cancer. Pneumologia. 2015;64:14–19. 26506668

[24] Binder-Foucard F, Bossard N, Delafosse P et al. Cancer incidence and mortality in France over the 1980–2012 period: solid tumors. Rev Epidemiol Sante Publique. 2014;62:95–108. 10.1016/j.respe.2013.11.073 24613140

[25] Cambier JF, Ronco P. Onco-nephrology: glomerular diseases with cancer. Clin J Am Soc Nephrol. 2012;7:1701–12. 10.2215/CJN.03770412 22904123

[26] Grimbert P, Valanciute A, Audard V et al. Truncation of C-mip (Tc-mip), a new proximal signaling protein, induces c-maf Th2 transcription factor and cytoskeleton reorganization. J Exp Med. 2003;198:797–807. 10.1084/jem.20030566 PMC1865475 12939343

[27] Sendeyo K, Audard V, Zhang S-Y et al. Upregulation of c-mip is closely related to podocyte dysfunction in membranous nephropathy. Kidney Int. 2013;83:414–25. 10.1038/ki.2012.426 PMC3927461 23302718

[28] Bouachi K, Moktefi A, Zhang S-Y et al. Expression of CMIP in podocytes is restricted to specific classes of lupus nephritis. PLoS One. 2018;13:e0207066. 10.1371/journal.pone.0207066 PMC6237342 30439969

[29] Liu Y, Su L, Lin Q et al. Induction of c-mip by IL-17 plays an important role in adriamycin-induced podocyte damage. Cell Physiol Biochem. 2015;36:1274–90. 10.1159/000430296 26160339

[30] Furuto Y, Hashimoto H, Namikawa A et al. Focal segmental glomerulosclerosis lesion associated with inhibition of tyrosine kinases by lenvatinib: a case report. BMC Nephrol. 2018;19:273. 10.1186/s12882-018-1074-3 PMC6194623 30340546

[31] Zhang SY, Kamal M, Dahan K et al. c-mip impairs podocyte proximal signaling and induces heavy proteinuria. Sci Signal. 2010;3:ra39. 10.1126/scisignal.2000678 PMC2897853 20484117

[32] Oniszczuk J, Sendeyo K, Chhuon C et al. CMIP is a negative regulator of T cell signaling. Cell Mol Immunol. 2020;17:1026–41. 10.1038/s41423-019-0266-5 PMC7609264 31395948

[33] Kamal M, Pawlak A, BenMohamed F et al. C-mip interacts with the p85 subunit of PI3 kinase and exerts a dual effect on ERK signaling via the recruitment of Dip1 and DAP kinase. FEBS Lett. 2010;584:500–6. 10.1016/j.febslet.2009.12.015 20018188

[34] Ollero M, Sahali D. The enigmatic emerging role of the C-Maf inducing protein in cancer. Diagnostics (Basel). 2021;11:666. 10.3390/diagnostics11040666 PMC8068179 33917766

