# Supplementary material for: Paraneoplastic podocytopathies with nephrotic syndrome in solid cancers with C-Maf inducing protein expression
Source: Clin Kidney J. 2026 Oct 15;19(11):sfag370. doi: 10.1093/ckj/sfag370 (PMC13748979; doi:10.1093/ckj/sfag370)
Supplement: Supplementary file 1 [file sfag370_supplemental_file.docx]

**Online supplemental material**

Table of contents:

- Supplemental methods – p2
- Supplemental Figure – p3
  - Supplemental Figure 1– Specificity assays for the anti-CMIP antibody – p3
  - Supplemental Figure 2. Representative double immunofluorescence staining for CD8/CMIP, and CD20/CMIP, analyzed by confocal microscopy in samples from patients with paraneoplastic nephrotic syndrome associated with cancer (PPNS) and control (CON). – p5

**Detailled methods (immunohistochemistry and confocal microscopy)**

For the immunohistochemistry study, paraffin-embedded tissue sections were dewaxed and rehydrated. Antigen retrieval was performed by immersing slides in boiling 0.01M citrate buffer in a 500W microwave oven for 15 minutes. Endogenous peroxidase activity was blocked with 0.3% H2O2 in methanol for 30 minutes. Slides were incubated with blocking reagents containing avidin-biotin solution for 30 minutes and normal blocking serum for 20 minutes. The CMIP polyclonal antibody was produced in rabbits immunized against peptides located in N-terminal (Pleckstrin Homology domain) and C-terminal (leucin rich repeat domain) of CMIP protein 15. Tissue sections were incubated overnight at 4°C with anti-CMIP antibody at a final concentration of 2.5 μg/mL. Rabbit IgG isotype control (Biocare Medical) was used instead of primary antibody as negative control. Slides were washed several times with TBS-Tween 0,1% for 60 min, then incubated with biotinylated secondary antibody for 60 min. Staining was visualized by incubation with Avidin-biotinylated horseradish peroxidase complex (Vectastain ABC Reagent; Vector Laboratories) with 3,3’-diaminobenzidine (Sigma-Aldrich). Slides were then counterstained with hematoxylin.

For immunofluorescence on paraffin sections, the primary antibodies used in this study include monoclonal rabbit against CD3E (#A700-016), CD8A (#A700-044), and FOXP3 (#A700-034) from Fortis (Fortis life sciences/BETHYL Laboratories, Inc), rabbit anti-CD4 (ab133616) and anti-PD-1 (ab237728) from Abcam, anti-CD20 (#A48750) (Cell Signaling Technology). The mouse monoclonal anti-CMIP (8D5) was developed by our laboratory. After dewaxing, rehydration and antigen retrieval as described above, paraffin sections were blocked in 5% BSA-TBS for 1 hour at room temperature, then incubated for 10 min at room temperature (RT) with the Fc receptor blocking solution TruStain Fc-X(TM) (BioLegend). Indicated antibodies were stained on these sections at 4°C overnight. After washing with PBS, slides were simultaneously incubated with Alexa-555-conjugated donkey anti-rabbit IgG and alexia-488 goat anti-mouse IgG (Molecular Probes, 8µg/ml in PBS, 50µl/slide), then covered with Vectashield mounting containing DAPI (4′,6-diamidino-2-phenylindole) (Vector Laboratories). Negative controls were stained with universal IgG isotype control (rabbit or MOPC 21) instead of primary antibodies. Tissue sections were imaged by a confocal laser scanning microscope LSM510-META (Carl Zeiss, Germany). For quantification, all glomeruli from each section, except those with significant sclerosis were analyzed by computer-assisted image analysis using X400 magnification. The images were blindly analyzed by two independent investigators. Positive staining within each glomerulus was expressed as the percentage of the immunostained area over the total glomerular area using the image analysis software (Image J; National Institute of Health, Bethesda, MD).

**Supplemental Figure 1. Specificity assays for the anti-CMIP antibody**

Immunostaining of CD4 T cells isolated by negative immunoselection using the mouse CD4^+^ T Cell Isolation Kit

(Miltenyi). CD4 T cells from wild-type mice and CMIP transgenic mice (a mouse model previously reported : Cellular Molecular Immunology, 17(10):1026-1041.2019) were co-labelled with Anti-CD4 (red) and anti-CMIP monoclonal antibody (Mab 8D5) or a non-specific Mab (MOPC 21) (green) , then analyzed by confocal microscopy. Scale bar 10 μm.

**A**

Western blot of Mab 8D5 on protein lysates from HEK cells transfected with human Cmip expression plasmid containing a V5 tag). Positive (anti-V5 Mab) and negative control (MOPC 21) were included.

**B**

**Supplemental Figure 2. Representative double immunofluorescence staining for CD8/CMIP, and CD20/CMIP, analyzed by confocal microscopy in samples from patients with paraneoplastic nephrotic syndrome associated with cancer (PPNS) and control (CON).** Scale bar = 20 µm.
